# The Devil Is in the Data: Can Regional Variation in Amenable Mortality Help to Understand Changes in Health System Performance in Poland?

**DOI:** 10.3390/ijerph19074129

**Published:** 2022-03-31

**Authors:** Anna Sagan, Marina Karanikolos, Małgorzata Gałązka-Sobotka, Martin McKee, Monika Rozkrut, Iwona Kowalska-Bobko

**Affiliations:** 1 European Observatory on Health Systems and Policies, London School of Economics and Political Science, London WC2A 2AE, UK; 2 European Observatory on Health Systems and Policies, London School of Hygiene & Tropical Medicine, London WC1H 9SH, UK; marina.karanikolos@lshtm.ac.uk (M.K.); martin.mckee@lshtm.ac.uk (M.M.); 3 Institute of Healthcare Management, Faculty of Economics and Management, Lazarski University, 02-662 Warszawa, Poland; m.galazka-sobotka@lazarski.edu.pl; 4 Department of Econometrics and Statistics, Institute of Economics and Finance, University of Szczecin, 70-453 Szczecin, Poland; monika.rozkrut@usz.edu.pl; 5 Institute of Public Health, Faculty of Health Sciences, Jagiellonian University, 31-007 Kraków, Poland; iw.kowalska@uj.edu.pl

**Keywords:** amenable mortality, health system, healthcare, public health, Poland

## Abstract

The contribution of health systems to health is commonly assessed using levels of amenable mortality. Few such studies exist for Poland, with analyses of within-the-country patterns being particularly scarce. The aim of this paper is to analyse differences in amenable mortality levels and trends across Poland’s regions using the most recent data and to gain a more nuanced understanding of these differences and possible reasons behind them. This can inform future health policy decisions, particularly when it comes to efforts to improve health system performance. We used national and regional mortality data to construct amenable mortality rates between 2002 and 2019. We found that the initially observed decline in amenable mortality stagnated between 2014 and 2019, something not seen elsewhere in Europe. The main driver behind this trend is the change in ischemic heart disease (IHD) mortality. However, we also found that there is a systematic underreporting of IHD as a cause of death in Poland in favour of heart failure, which makes analysis of health system performance using amenable mortality as an indicator less reliable. We also found substantial geographical differences in amenable mortality levels and trends across Poland, which ranged from −3.3% to +8.1% across the regions in 2014–2019. These are much bigger than variations in total mortality trends, ranging from −1.5% to −0.2% in the same period, which suggests that quality of care across regions varies substantially, although some of this effect is also a coding artefact. This means that interpretation of health system performance indicators is not straightforward and may prevent implementation of policies that are needed to improve population health.

## 1. Introduction

As with its neighbours in Eastern Europe, life expectancy (LE) in Poland increased rapidly in the 1990s and 2000s [1,2]. Yet this was not enough to close the gap with the pre-2004 European Union (EU) countries; by 2019, life expectancy at birth (both sexes) was still more than three years lower, with no sign of narrowing [1,3]. The reasons for the initial improvement have been studied extensively. The early work focused on the link between social and economic changes post-transition and a rapid decline in deaths from cardio-vascular disease (although interrupted by a brief increase in deaths from external causes) [4,5,6], soon complemented by research on the contribution of a reformed health system [7]. This more recent body of work mostly looked at Poland as a whole, typically comparing it with its neighbours, [8,9], with a few studies of patterns within the country [7,10]. The latter use data that are at least a decade old and do not capture the most recent trends and/or focus on more granular geographical variations in amenable mortality, which may be more reflective of primary healthcare (PHC) provision.

The contribution of health systems to health is commonly assessed using levels of amenable mortality, as in the studies cited above. The measure captures deaths that should not occur in the presence of timely and effective care [11,12] and is incorporated in the Global Burden of Disease (GBD) Health Access and Quality Index, and the Universal Health Coverage Index of effective health services, with the latter showing that, in 2019, Poland lagged behind many EU countries in quality of care [13,14]. An earlier study that analysed the decline in amenable mortality in Organisation for Economic Co-operation and Development (OECD) countries between 2000 and 2014 found that the pace of improvement in Poland was similar to that in other countries of eastern Europe, which in the early 2000s started off at a similar level (e.g., the Czech Republic) [15], but stagnated during the five years pre-dating the COVID-19 pandemic (2014–2019), something not seen elsewhere in the EU [16].

This situation has arisen despite the efforts, over more than two decades, by successive Polish governments to reform the health system. Consistent elements have included measures to tackle the high burden of non-communicable diseases (NCDs), mainly cardiovascular diseases and cancers, strengthening primary care by transferring responsibilities from the very large hospital sector inherited from the Communist period, and taking measures to improve quality, accessibility, and continuity [17,18,19]. Yet despite having ambitious goals for reform, in 2019 spending on health remained well below that in other EU countries, at USD 2207 (adjusted for purchasing power) per capita [20]. For comparison, the figures for Germany and France were USD 6739 and USD 5493, respectively.

The earlier finding of large geographical inequalities in several measures of quality of care within Poland, including amenable mortality [10], and the recent arrest in the previous improvements, point to the need to understand the situation at a sub-national level. The aim of this paper is to analyse the recent lack of improvement in amenable mortality in Poland’s regions and to gain a more nuanced understanding of the trends to inform health policy decisions, particularly when it comes to the efforts to improve health system performance. By focusing on regional variations, we can gain insight into timeliness and effectiveness of not only primary healthcare (PHC), but also other healthcare services, and into broader national and regional policies aimed at addressing amenable mortality.

## 2. Materials and Methods

We extracted data on mortality by region and cause of death according to the International Classification of Diseases, Tenth Revision (ICD-10) from the demographic database of the Chief Statistical Office in Poland [21], and population size from the Local Data Bank of the Chief Statistical Office in Poland [22] complemented with demographic data from Eurostat [16]. We constructed age-standardised mortality rates from 2002 to 2019 (years with complete available data at the time of the analysis as of August 2021). These were standardised to European Standard Population 2013 [23], which reflects the most recent EU population standard.

Amenable mortality is defined as deaths that should not occur in the presence of timely and effective healthcare [12]. We used the list of amenable causes of death compiled by Nolte and McKee [12] (see Table S1 in the Supplementary Materials), which considers only 50% of deaths from ischemic heart disease as amenable. The upper age cut-off for both amenable and total mortality in this study was set at 75 years of age. The year 2014 was identified as when the slowdown in both total and amenable mortality became noticeable nationally. We estimated the average annual percentage change (AAPC) in mortality for the periods before and after this year (2002–2014 and 2014–2019) using Microsoft Excel. We supplemented the analysis of change in amenable mortality by disaggregating cardio-vascular diseases into more specific causes to understand the key drivers behind the trends.

## 3. Results

**Figure 1:**
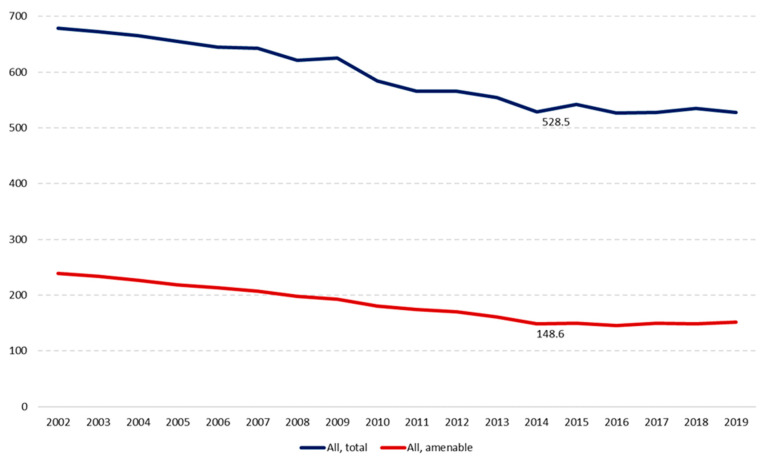
Trends in mortality in Poland, 2002–2019, age-standardised rate per 100,000 population.

Figure 1 shows how, between 2002 and 2019, mortality from all causes in people aged under 75 in Poland decreased from 679 to 528 per 100,000 (AAPC = −1.44%). In the same period, amenable mortality decreased faster, from 239 to 152 per 100,000 (AAPC = −2.58%). However, although between 2002 and 2014 the contribution of amenable mortality to total deaths decreased between 2002 and 2014 from 35% of the total for this age group to 28%, it had slightly grown (to 29%) by 2019.

When we look at the two periods we can see how, between 2002 and 2014, mortality from all causes in those under the age of 75 in Poland had been decreasing annually on average by 2.0%, while the pace of decrease in amenable mortality was twice as fast, at 3.9%. However, between 2014 and 2019, the pace of decline in total and amenable deaths slowed to 0.8% and 0.9% respectively, meaning the relative progress in amenable mortality was almost four times slower than in the preceding years.

**Figure 2:**
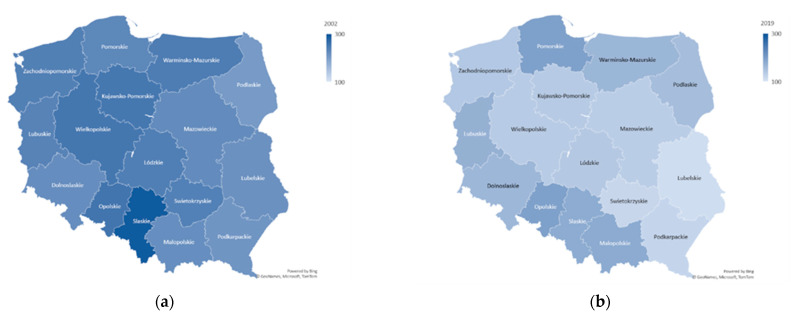
Distribution of amenable mortality rates in Polish regions in 2002 (**a**) and 2019 (**b**).

We now turn to the regional picture within Poland. Figure 2 shows the distribution of amenable mortality rates in Polish regions in 2002 and 2019, revealing an improvement everywhere but with the highest rates in some of the southern regions in both periods.

**Figure 3:**
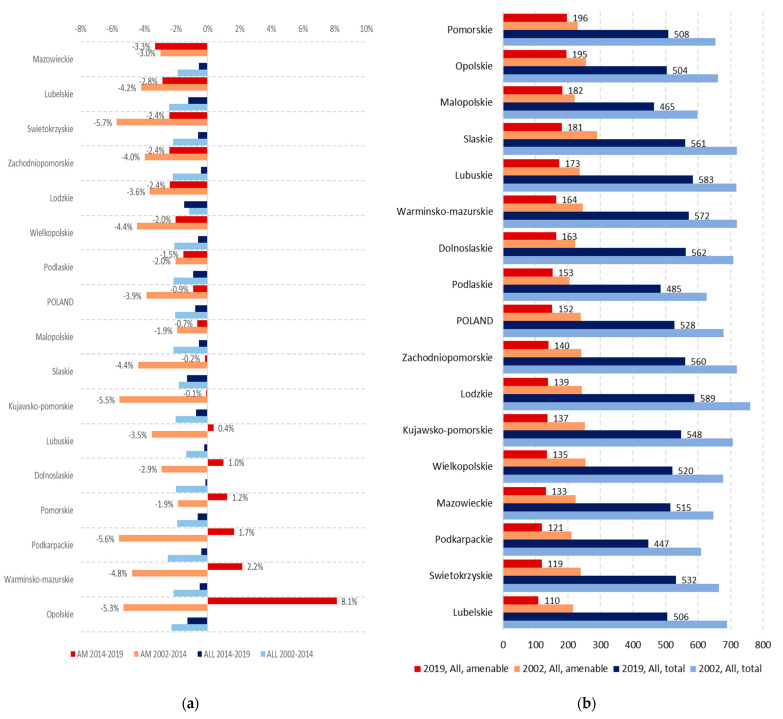
Total and amenable mortality, 2002–2019: (**a**) change in total and amenable mortality (AAPC, both sexes) by region in 2002–2014 and 2014–2019 (data labels only shown for amenable mortality for reasons of space); (**b**) total and amenable mortality (standardised rates per 100,000, both sexes) by region in 2002 and 2019.

Figure 3a looks at the changes in more detail. Looking first at amenable mortality, in 2002–2014 the pace of decrease varied from AAPC = −5.7% in Swietokrzyskie to AAPC = −1.9% in Malopolskie (the region that contains Warsaw). However, in 2014–2019 the pace of change slowed and even reversed in some regions, ranging from AAPC = −3.3% in Mazowieckie, to an increase in AAPC of 8.1% in Opolskie, Poland’s smallest and least populated region. Mazowieckie also stands out as the only region where the pace of reduction in amenable mortality was not only maintained, but also improved in 2014–2019. Notably, in addition to the Opolskie region, there were five more regions where amenable mortality increased between 2014 and 2019, and a further two where it stagnated, with AAPC less than −0.2%. Overall, between 2002 and 2019, amenable mortality fell by over 40% in eight regions (Swietokrzyskie, Lubelskie, Wielkopolskie, Kujawsko-Pomorskie, Podkarpackie, Lodzkie, Zachodniopomorskie, and Mazowieckie) (Figure 3b). These regions recorded the lowest rates of amenable mortality in 2019, which ranged from 110 per 100,000 population in Lubelskie to 196 in Pomorskie, a difference of almost 80%.

There was much less variation in the pace of change in overall mortality across regions in both periods (from AAPC = −2.5% in Podkarpackie to AAPC = −1.2% in Lodzkie in 2002–2014, and from AAPC = −1.5% in Lodzkie to AAPC = −0.2% in Dolnoslaskie) (Figure 2a). In contrast to amenable mortality, no region saw a reversal in the decline in total mortality in 2014–2019 (Figure 3b). Despite the reductions in amenable mortality, Lodzkie still had the highest total mortality rate in 2019 (589 per 100,000) and total mortality rates in some of the other regions that observed high reductions in amenable mortality (Zachodniopomorskie, Kujawsko-Pomorskie, and Swietokrzyskie) were also among the highest in Poland (Figure 3b). At 447 deaths per 100,000 population, Podkarpackie had the lowest total mortality rate in Poland in 2019.

Table S2 in the Supplementary Materials shows AAPC in amenable mortality in all of Poland and in each region for the two periods by major cause of amenable mortality (ischemic heart disease (IHD), cancer, stroke, respiratory disease, and aggregated group of other (remaining) amenable causes). In Poland overall, the rate of decline in IHD slowed markedly (from AAPC = −5.29% in 2002–2014 to AAPC = −0.25% in 2014–2019). The change in IHD (where 50% deaths are considered amenable) was the main driver of the trend in amenable mortality, accounting for 32% of all amenable causes in 2019. Cancer was the second major contributor and accounted for 23% of all amenable deaths. This was the only category of amenable deaths that saw an accelerating decline—from AAPC = −0.80% in 2002–2014 to AAPC = −0.97% in 2014–2019. Stroke was the third major contributor in 2019, accounting for 19% of amenable deaths. As with IHD, the pace of improvement in mortality from stroke has slowed in more recent years—from AAPC = −5.05% to AAPC = −2.95%. Respiratory conditions were responsible for 12% of amenable deaths and, unlike with other causes, the trend here has been upwards throughout the period, with an AAPC increase of 3.55% in 2002–2014, doubling to AAPC 8.87%. In absolute terms, standardised mortality rate from amenable respiratory conditions increased from 8 to 18 per 100,000 population. Other amenable causes (aggregated group containing conditions such as infectious diseases, digestive disorders, perinatal and congenital conditions, diabetes, and others) accounted for the remaining 15%. This group experienced a decline of AAPC = −4.91% in 2002–2014, and an increase of AAPC = 4.96% in 2014–2019.

Turning to the 16 Polish regions, there was much more geographical variation with amenable causes in the second period, in both levels and rate of change. For example, while in the Opolskie region the reduction in amenable IHD deaths was initially very rapid, at AAPC = −8.32%, the reversal in 2014–2019 amounted to a striking increase of AAPC = 22.99%. Changes in IHD in other regions were less drastic, but notable reversals of earlier progress were seen in Dolnoslaskie, Lubuskie, Slaskie, Swietokrzyskie, and Warminsko-Mazurskie. Interestingly, in the Mazowieckie region, amenable IHD deaths continued to decline even more rapidly—from AAPC = −3.86% to AAPC = −9.65% in 2002–2014 and 2014–2019, respectively. Trends in stroke showed similar patterns, although with fewer reversals and, where they occurred, on a much smaller scale. The Opolskie region still stood out in terms of experiencing one of the fastest initial improvements that turned into a reversal with one of the greatest magnitudes. Not all regions saw a decline in the AAPC for amenable cancer deaths in 2002–2014. For example, in Lubuskie and Opolskie, amenable mortality from cancer was increasing. In both regions, however, it started declining in 2014–2019. In contrast, in Dalnoslaskie, Lodzkie, Lubelskie and Warminsko-Mazurskie, the initial decline was reversed in the later period. All regions showed a sustained increase in amenable respiratory deaths, which was usually faster in the latter period.

Given the increasing differences among regions, we analysed mortality from cardiovascular diseases in more detail. This reveals an important issue with coding causes of death. In the majority (10/16) of Polish regions (Dolnoslaskie, Kujawsko-Pomorskie, Lodzkie, Lubelskie, Lubuskie, Mazowieckie, Podkarpackie, Swietokrzyskie, Wielkopolskie, and Zachodniopomorskie), the fall in the numbers of registered IHD deaths corresponds to the rise in recorded deaths from heart failure (a non-amenable cause). In contrast, Malopolskie, Podlaskie, Pomorskie, Slaskie, and Warminsko-Mazurskie regions recorded a very different pattern for most of the period, with low numbers of heart failure deaths and the numbers of IHD deaths remaining consistently high and/or increasing in more recent years. The Opolskie region stands out in terms of irregularity of recording of both IHD and heart failure deaths, with a drop in IHD from 918 to 192 cases between 2000 and 2016 followed by sharp rise to 857 deaths in 2019. The latter rise drives the increase in the amenable mortality indicator since 2014 shown in Figure 3a. Overall, these regional patterns of transfers between causes of death create a picture of lack of progress in reducing IHD deaths and amenable mortality since 2014 (Figure S1 in the upplementary Materials). There is a systematic under-reporting of IHD as a cause of death in Poland (Figure S2 in the Supplementary Materials), which, by implication, makes analysis of health system performance using amenable mortality as an indicator less reliable. The lack of accurate information may affect health policies and result in de-prioritisation of action on prevention and treatment of IHD.

## 4. Discussion

Amenable mortality kept falling in Poland between 2002 and 2019, continuing the trend observed in earlier studies [24,25]. Broadly, and almost universally, amenable mortality declined more slowly since 2014, but in some regions it even reversed. This was driven, at least in part, by changes in recording cause of death. Below we discuss some possible explanations.

First, there was a slowdown in progress in terms of reducing deaths from IHD and stroke, with amenable deaths from these causes seeing much smaller reductions after 2014. Previous studies attributed the positive trends in cardiovascular diseases in 2000–2014 to social and economic changes that led to positive lifestyle changes, in addition to improvements in the health system, including implementation of advanced treatment methods [26]. Bandosz and colleagues (2012), who looked at mortality trends in coronary heart disease between 1991 and 2005, found that about 37% of the decrease observed over this period was attributable to increased uptake of evidence-based treatments, including treatments for hypercholesterolaemia, hypertension, coronary heart disease, and heart failure, in addition to coronary bypass surgery, coronary angioplasty, and stenting, with 54% of the trend explained by changes in risk factors, mainly resulting from improved diets and physical activity [27]. Investment in cardiac care, including dedicated national preventive and curative programmes, increased by more than three-fold between 2004 and 2014 [28]. As a result, both access to acute cardiological care (e.g., increasing centres offering invasive cardiology) and outcomes (e.g., the 30-day mortality rate after hospital admission for acute myocardial infarction (AMI) fell to 4.4 per 100 admissions compared to the OECD average of 7.5) improved, and this means that Poland is now regarded as one of European leaders in invasive treatment of acute coronary syndromes (ACS) [29]. The reason for the slower pace in the decrease in amenable mortality from IHD observed in recent years may be because some of the potential improvements in cardiac care may have already been realised and it is now harder to achieve further gains. However, there remain deficiencies in the provision of primary and specialist outpatient cardiac care, particularly cardiac rehabilitation, in addition to primary and secondary prevention. Less than a quarter of Polish patients undergo rehabilitation after myocardial infarction, compared to 30–50% in Western Europe [29]. If these deficiencies were addressed, further reductions in amenable mortality may possibly be achieved. The introduction, in 2017, of a complex care programme for patients after myocardial infarction that focuses on rehabilitation has the potential to further improve patient outcomes and has already shown positive early results [29]. Furthermore, more recent trends in IHD mortality are less reliable, due to coding practices [30].

Another major cause of amenable deaths in Poland is stroke. In 2005, 30-day hospital fatality among patients treated for cerebral haemorrhage, at 36.9%, was one of the highest in the OECD [31]. Despite the creation of a network of stroke units within the National Health Programme 2007–2015, which achieved lower morality rates than in general wards, and the increasing share of stroke patients hospitalised in these centres, fatality rates remain high. This can be linked to poor access to these units (with between 53% and 84% of patients with ischemic stroke being admitted in different regions), treatment such as thrombolytic treatment (which was provided to only 13% of hospitalised stroke patients) or transurethral mechanical thrombectomy of intracerebral or intracranial vessels (which was only introduced in December 2018 via a pilot programme), and rehabilitation (only 24% of stroke ward patients and 15% of other stroke patients received rehabilitation within 14 days from discharge) [32]. Hospital fatality with haemorrhagic stroke deteriorated in 2008–2013 and then again from 2017 (back to the 2012 level), with a brief period of improvement since 2014. The hospital fatality rate for ischemic stroke decreased very slowly—by 3% between 2004 and 2018. Fatality for unspecified stroke fell to 14.3% in 2018 from 23.7% in 2004. The reasons for the slowdown in the decline in the amenable mortality from stroke may reflect rising incidence rates, improved diagnosis and detection, and low rates of post-stroke rehabilitation. In 2017, only about one-fifth of patients started rehabilitation within 14 days of hospital discharge [32].

Third, there was a small but sustained improvement in outcomes for treatable cancers in 2014–2019. This occurred at the same time as the so-called ‘fast oncology pathway’ was introduced in January 2015, one element of which involved abolishing financing limits for all services provided within this programme. As a result, total spending on cancer care increased by over 35% between 2014 and 2019 [33]. The reason why this improvement has been small may be due to the programme not being homogeneously implemented at all levels of care. For example, cancer screening rates remain low, and cancers are detected at a relatively advanced stage [34] with, for example, 40% of newly diagnosed cases of cervical cancer being diagnosed too late for successful treatment [35]. Moreover, only 35% of referrals to the ‘fast pathway’ were from primary providers between 2018 and 2020 [36]. Improvements in waiting times for diagnostics and treatment for patients within the pathway have also been negligible (and appear to have worsened for patients not included in the pathway) [37]. Further, the implementation of the pathway has led to increased fragmentation in the provision of cancer care, which combined with the lack of reference levels and standardised guidelines for diagnostics and treatment, can result in unequal quality of care [37]. The introduction, in 2019, of the National Oncology Network, currently being piloted in four regions, has the potential to address these shortcomings and improve patient outcomes in the years to come.

Fourth, there was a continuous rise in amenable mortality from respiratory conditions, especially pneumonia, in 2014–2019. This is contrary to the trend observed in the majority of countries in Europe [38]. High levels of air pollution in Poland—which are the highest in the EU [31]—have been linked to increased respiratory diseases, including bronchitis, asthma, rhinitis, and lung cancer [39]. In addition to air pollution control and other preventive measures, such as smoking cessation campaigns or pneumococcal and influenza vaccination strategies, health care interventions such as more appropriate use of antibiotics and improved medical care can lead to a decrease in pneumonia mortality [38]. In Poland, as in other countries in Central and Eastern Europe, mortality in patients hospitalised due to community-acquired pneumonia is twice as high as in Western Europe [40]. This has been linked to the specific use of antibiotics such as aminopenicillins and concerns about antimicrobial resistance (Poland has one of the highest rates of antibiotics consumption in Europe [41]), in addition to lower standards of diagnostics and care [40,42]. Deficiencies in the treatment of other respiratory diseases, such as asthma, have also been documented [43,44,45].

Finally, there was a reversal in the aggregate ‘other amenable causes’ group during 2014–2019, with amenable mortality increasing in this period. However, the absolute rates for individual conditions in the group remain small, particularly for the population aged under 75, and may be subject to random fluctuations.

It is beyond the scope of this paper to determine the reasons for the regional variation in amenable mortality. However, we can point to some likely factors. For example, the numbers of physicians, nurses, and items of advanced medical equipment are the highest in Mazowieckie region, containing Warsaw, while they are among the lowest in Opolskie [46]. Uptake of complex care programmes such as the one for patients after AMI (introduced in 2017) [47] or the one focusing on strengthening PHC [48] also varies among the regions, as does the number of stroke wards [32]. Lodzkie requires specific attention, as the life expectancy of men living in this region has been consistently and markedly lower than that in the other regions, and at 72.5 years in 2019 was 2.9 years shorter than that in Podlaskie, where male life expectancy was the highest [31]. These worse outcomes have been attributed to unhealthy lifestyles [26,49,50].

The study is subject to several limitations. In addition to the well-described limitations of using amenable mortality as an indicator of health system performance (focus on mortality as an outcome, restricted age, and selection of causes considered amenable), the indicator is heavily reliant on accurate recording of deaths. Poland stands out among other countries in Europe in terms of the very high number of ‘garbage codes’ (codes that are not useful in terms of determining public health trends in mortality outcomes, and that may hinder international comparisons), among which a particularly large share is coded to heart failure and generalised and unspecified atherosclerosis (I50 and I70.9 respectively in the ICD−10 classification). Fihel and Muszyńska-Spielauer [30] find that, in Poland, more than one-fifth (22%) of total deaths in 2013 were initially assigned to one of the ‘garbage codes’ and, after re-classification using original death records and coarsened exact matching, the age-standardised death rate for IHD increased by 43%, and the rate for stroke by 22%. The use of garbage codes increases sharply with age; therefore, the age cut-off at 75 years partially mitigates this problem. However, as demonstrated in Figure S2 in Supplementary Materials, patterns of recording of IHD and heart failure in those aged under 75 make a big impact on trends and scale for amenable mortality as a whole, suggesting that some of the observed patterns are a coding artefact. While an in-depth investigation of reasons for the observed patterns of recording cause of death is beyond the scope of this paper, the quality of hospital coding has often been raised [51]. With many hospitals struggling to balance their budgets [52,53], recording heart failure potentially attracts larger payments in the Polish diagnosis-related group (DRG) system and distorts coding of deaths.

## 5. Conclusions

National or cross-country analyses of mortality can mask marked within-country differences that are important for policy. In line with previous studies linking amenable mortality to healthcare, this paper reveals substantial geographical differences in amenable mortality levels and trends across Poland. These are much bigger than variations in total mortality, which suggest that quality of care across regions varies substantially. However, some of the observed patterns are due to poor recording of deaths and distortion of patterns of amenable mortality by underestimating the burden of IHD. This, in turn, can hinder interpretation of health system performance indicators and prevent implementation of policies to improve population health. Policy makers in Poland should thus focus on improving the quality of amenable mortality data both going forwards, which includes addressing the drivers of poor recording of deaths, such as adjusting the costing of DRGs, but also retrospectively, by ensuring harmonisation of past data across the regions and years. Future research work can then focus on establishing meaningful statistical associations between potential drivers of the variations in amenable mortality across the region. Such analyses and more accurate data on amenable mortality would support efforts to improve access and quality of care for conditions that are amenable to healthcare, in addition to the health system performance more broadly.

## Data Availability

Data supporting reported results can be obtained from the authors on request.

## Supplementary Materials

The following are available online at https://www.mdpi.com/article/10.3390/ijerph19074129/s1, Figure S1: Number of deaths due to ischemic heart disease and heart failure in Poland, 2000–2019, Figure S2: Number of deaths due to ischemic heart disease and heart failure in Polish regions, 2000–2019, Table S1: Causes of death considered amenable to health care, Table S2: Change in amenable mortality by major cause of death, 2002–2019.
