# Supplementary material for: The Devil Is in the Data: Can Regional Variation in Amenable Mortality Help to Understand Changes in Health System Performance in Poland?
Source: Int J Environ Res Public Health. 2022 Mar 31;19(7):4129. doi: 10.3390/ijerph19074129 (PMC8998952; doi:10.3390/ijerph19074129)
Supplement: Supplementary file 1 [file ijerph-19-04129-s001.zip › ijerph-1640712-supplementary.pdf]

Supplementary Materials:

Table S1. Causes of death considered amenable to health care

	**Name of group**	**Age**	**ICD-10**
**1**	Intestinal infections	0-14	A00-A09
**2**	Tuberculosis	0-74	A15-A19, B90
**3**	Other infectious (Diphtheria, Tetanus, Poliomyelitis)	0-74	A36, A35, A80
**4**	Whooping cough	0-14	A37
**5**	Septicaemia	0-74	A40-A41
**6**	Measles	1-14	B05
**7**	Malignant neoplasm of colon and rectum	0-74	C18-C21
**8**	Malignant neoplasm of skin	0-74	C44
**9**	Malignant neoplasm of breast	0-74	C50
**10**	Malignant neoplasm of cervix uteri	0-74	C53
**11**	Malignant neoplasm of cervix uteri and body of the uterus	0-44	C54, C55
**12**	Malignant neoplasm of testis	0-74	C62
**13**	Hodgkin's disease	0-74	C81
**14**	Leukaemia	0-44	C91-C95
**15**	Diseases of the thyroid	0-74	E00-E07
**16**	Diabetes mellitus	0-49	E10-E14
**17**	Epilepsy	0-74	G40-G41
**18**	Chronic rheumatic heart disease	0-74	I05-I09
**19**	Hypertensive disease	0-74	I10-I13, I15
**20**	Ischaemic heart disease	0-74	I20-I25
**21**	Cerebrovascular disease	0-74	I60-I69
**22**	All respiratory diseases (excl. pneumonia/influenza)	1-14	J00-J09, J20-J99
**23**	Influenza	0-74	J10-J11
**24**	Pneumonia	0-74	J12-J18
**25**	Peptic ulcer	0-74	K25-K27
**26**	Appendicitis	0-74	K35-K38
**27**	Abdominal hernia	0-74	K40-K46
**28**	Cholelithiasis & cholecystitis	0-74	K80-K81
**29**	Nephritis and nephrosis	0-74	N00-N07, N17-N19<br>N25-N27
**30**	Benign prostatic hyperplasia	0-74	N40
**31**	Maternal deaths	All	O00-O99
**32**	Congenital cardiovascular anomalies	0-74	Q20-Q28
**33**	Perinatal deaths, all causes excluding stillbirths	All	P00-P96, A33, A34
**34**	Misadventures to patients during surgical and medical care	All	Y60-Y69, Y83-Y84

Source: [12]

Table S2. Change in amenable mortality by major cause of death, 2002-2019

	IHD		STROKE		CANCER		RESPIRATORY DISEASES		OTHER DISEASES	
	AAPC 2002-2014	AAPC 2014-2019	AAPC 2002-2014	AAPC 2014-2019	AAPC 2002-2014	AAPC 2014-2019	AAPC 2002-2014	AAPC 2014-2019	AAPC 2002-2014	AAPC 2014-2019
Dolnoslaskie	-4.41%	2.58%	-3.08%	-0.52%	-0.27%	0.26%	10.30%	10.71%	-3.72%	-0.83%
Kujawsko-pomorskie	-7.91%	-1.06%	-5.39%	-1.50%	-0.98%	-1.03%	2.57%	11.66%	-9.62%	30.80%
Lodzkie	-6.09%	-4.19%	-4.00%	-5.03%	-0.54%	1.03%	3.55%	7.38%	-4.96%	6.33%
Lubelskie	-6.69%	-4.69%	-4.08%	-7.48%	-0.20%	0.37%	4.12%	6.39%	-4.66%	-0.36%
Lubuskie	-4.47%	8.74%	-4.85%	1.22%	0.88%	-1.95%	8.62%	17.20%	-5.28%	24.61%
Malopolskie	-0.37%	1.38%	-6.05%	-2.49%	-0.84%	-2.56%	2.14%	22.50%	-2.21%	1.12%
Mazowieckie	-3.86%	-9.65%	-5.19%	-3.83%	-0.78%	-0.71%	5.50%	4.38%	-3.26%	4.60%
Opolskie	-8.32%	29.99%	-7.08%	6.01%	0.50%	-0.73%	2.92%	28.71%	0.04%	8.43%
Podkarpackie	-7.13%	1.68%	-5.09%	-2.04%	-0.75%	-0.50%	7.18%	18.20%	-7.92%	34.99%
Podlaskie	-0.47%	-0.54%	-3.62%	-6.42%	-1.12%	-0.99%	6.21%	10.42%	-4.33%	4.79%
Pomorskie	-1.15%	3.75%	-4.35%	-2.68%	-0.67%	-1.96%	8.72%	2.87%	-3.26%	7.88%
Slaskie	-6.45%	2.39%	-4.76%	-3.45%	-0.38%	-1.58%	1.38%	7.58%	-4.53%	9.71%
Swietokrzyskie	-8.41%	1.73%	-6.12%	-5.40%	-0.22%	-2.90%	2.80%	18.07%	-7.84%	18.73%
Warminsko-mazurskie	-7.16%	9.72%	-5.75%	0.22%	-1.23%	2.54%	0.09%	8.95%	-1.59%	5.08%
Wielkopolskie	-6.85%	-5.73%	-4.80%	0.10%	-1.08%	-0.46%	7.72%	6.12%	-5.44%	-2.81%
Zachodniopomorskie	-7.13%	-3.88%	-5.31%	1.07%	-0.08%	-1.73%	8.82%	8.88%	-1.96%	-0.23%
**POLAND**	**-5.29%**	**-0.25%**	**-5.05%**	**-2.95%**	**-0.80%**	**-0.97%**	**3.55%**	**8.72%**	**-4.91%**	**4.96%**

Figure S1. Number of deaths due to ischemic heart disease and heart failure in Poland, 2000-2019

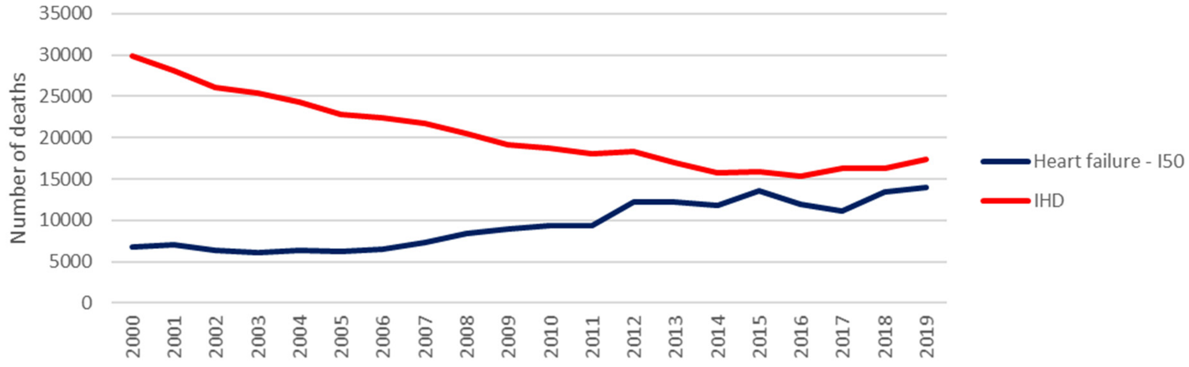

Figure S2. Number of deaths due to ischemic heart disease and heart failure in Polish regions, 2000-2019

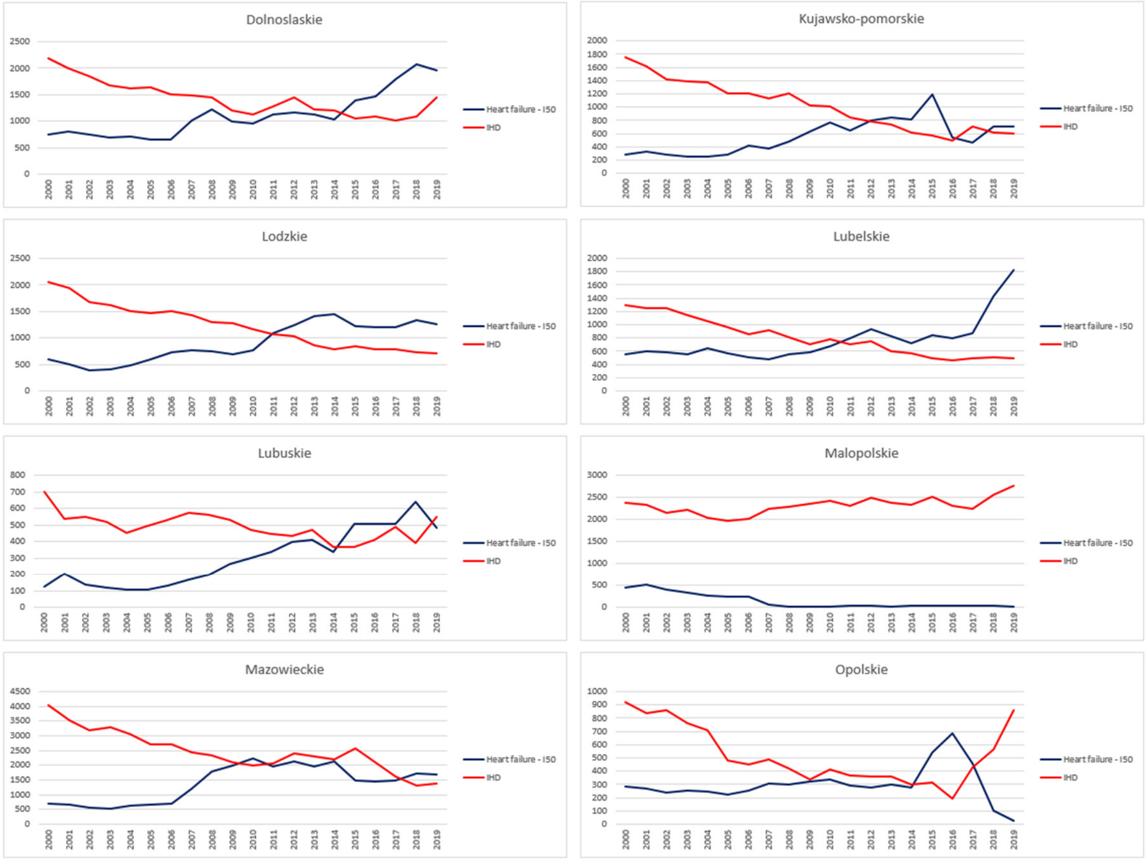

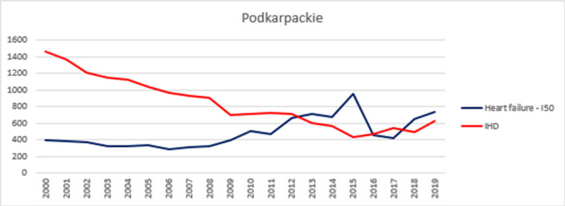
Podkarpackie
Heart failure - I50
IHD

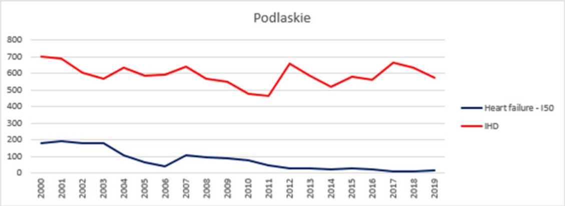
Podlaskie
Heart failure - I50
IHD

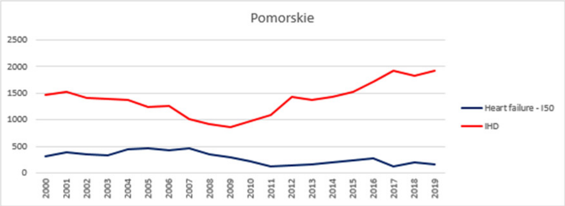
Pomorskie
Heart failure - I50
IHD

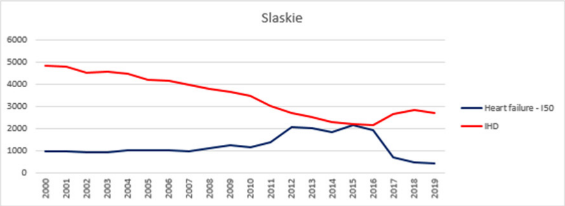
Slaskie
Heart failure - I50
IHD

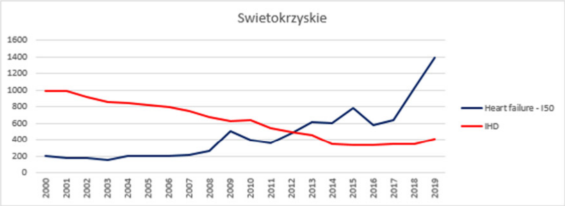
Swietokrzyskie
Heart failure - I50
IHD

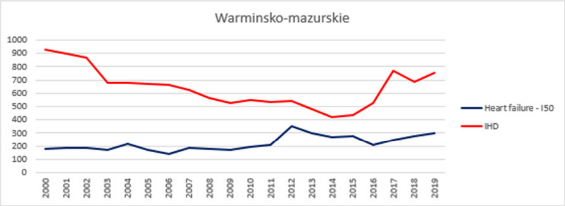
Warminsko-mazurskie
Heart failure - I50
IHD

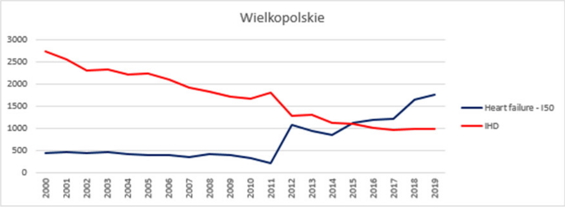
Wielkopolskie
Heart failure - I50
IHD

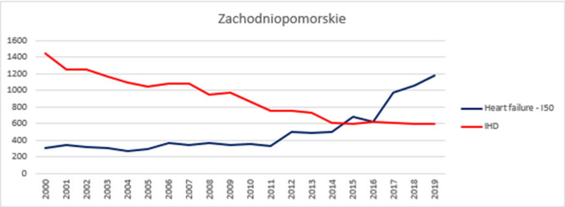
Zachodniopomorskie
Heart failure - I50
IHD
